# Clinical features of immunoglobulin G4-related spinal pachymeningitis

**DOI:** 10.3389/fnhum.2025.1541096

**Published:** 2025-07-17

**Authors:** Bingxue Zhang, Lenan Kang, Xiaohong Li

**Affiliations:** ^1^Department of Neurology, Dalian Friendship Hospital, Dalian, China; ^2^Department of Critical Care Medicine, The First Hospital of Qiqihaer, Qiqihar, China

**Keywords:** IgG4-related disease, IgG4-related disease (IgG4-RD), spinal pachymeningitis, clinical features, spinal, spinal cord injury

## 1 Introduction

Immunoglobulin G4 (IgG4)-related disease (IgG4-RD) is a systemic, chronic, inflammatory, and fibrotic condition related to the immune system that can affect various parts of the body (Al-Mujaini et al., 2018). However, neurological involvement is rare, particularly in reported cases involving the dura mater (Okazaki et al., 2011). Currently, both domestically and internationally, research on IgG4-related spinal pachymeningitis (IgG4-RSP) is mostly limited to case reports. There is a lack of research on the clinical features of this disease. This study retrospectively analyzed individual case reports of IgG4-RSP published in the PubMed, Web of Science, and Embase databases from 2009 to the present. The study then summarized and analyzed the clinical features of IgG4-RSP. The specific content is reported below.

## 2 Methods

### 2.1 Data sources

Using the search terms “immunoglobulin G4,” “hypertrophic spinal pachymeningitis,” “IgG4-related disease,” and “IgG4-related spinal pachymeningitis,” case reports and single-center studies published in the PubMed, Web of Science, and Embase databases were retrieved from domestic and international sources up to September 2024. A total of 362 articles were retrieved: 162 from PubMed, 98 from the Web of Science, and 102 from the Embase database. Of these, 140 were duplicates. After reading the full texts, irrelevant medical records and incomplete reports were excluded, resulting in 50 eligible articles comprising data on 55 patients. All patients were diagnosed with IgG4-RSP by a specialist clinician, and they had relatively complete clinical records. The articles were read in detail, and the following information was extracted: patient gender, age of onset, initial symptoms, clinical manifestations, laboratory tests, sites of lesions, pathological results, treatment methods, prognosis, and situations of misdiagnosis (Table 1).

**Table 1:** Clinical data of the 55 cases of IgG4-RSP.

**Study**	**Source**	**Gender**	**Age**	**Time**	**Initial symptom**	**Main symptoms**	**serum IgG4**	CSF cell count (0-8)^*^106	**CSF protein**	**CSF protein (mg/dl)**	**Involved area**	**MRIenhancement**	**Patterns ofenhancement**	**Pathology**	**IgG4+ cell**	**IgG4+/IgG**	**Treatment**	**Outcome**
2009	Chan et al.	Male	37	2w	Limb weakness	Limb weakness, loss of sensation, gait disorder	ND	ND	ND		T	Y	ND	Fibrosis inflammatory cell infiltration phlebitis	310	70	Decompression surgery	ND
2010	Choi et al.	Female	46	2w	Limb weakness	Limb weakness, loss of sensation, gait disorder	Normal	ND	ND		T	Y	Focal	Fibrosis inflammatory cell infiltration	20	ND	Decompression surgery Glucocorticoid	Improved
2012	Tajima et al.	Male	64	4w	Difficulty in swallowing	Difficulty in swallowing, hoarseness of voice, Muscle atrophy	Increase	14	Increase	260	B T	Y	Focal	Inflammatory cell infiltration	ND	ND	Glucocorticoid	Improved
2012	Della-Torre et al.	Male	65	48w	Headaches	Headaches, Muscle atrophy	Normal	1	Increase	82	B C	Y	Linear	Fibrosis inflammatory cell infiltration	ND	40-70	Glucocorticoid Methotrexate	Improved
2013	Wallace et al.	Male	32	ND	Pain Sensory changes and weakness of the limb	Pain, Sensory changes and weakness of the limb	ND	ND	ND		L	N	ND	Fibrosis inflammatory cell infiltration phlebitis	11	35	Decompression surgery	Improved
2013	Della-Torre et al.	Female	48	ND	Peripheral facial nerve palsy headaches	Headaches, facial nerve palsy, dizziness Trigeminal neuralgia, deafness difficulty in swallowing	ND	2	Normal	44	B C	Y	ND	Nd	ND	ND	Glucocorticoid Cyclophosphamide	Improved
2014	Sakai et al.	Female	32	24w	Headaches	Headaches, cervicalgia, limb weakness, urinary dysfunction, decline in vision acuity	Increase	30	Increase	91	B C L S	Y	Linear	Fibrosis inflammatory cell infiltration	ND	ND	Glucocorticoid	Improved
2014	Chen et al.	Male	49	48w	Doraslgia and leg pain	Doraslgia and leg pain, Limb weakness and loss of sensation	Increase	ND	ND		T L	Y	Focal	Fibrosis inflammatory cell infiltration	ND	ND	Decompression surgery Glucocorticoid	Improved
2014	Kim et al.	Female	52	2d	Limb weakness	Limb weakness and loss of sensation, Urinary dysfunction	ND	150	Increase	865	C T	Y	Focal	Fibrosis inflammatory cell infiltration	ND	ND	Decompression surgery Glucocorticoid	Improved
2014	Ezzeldin et al.	Male	55	2w	Limb weakness	Limb weakness and loss of sensation, gait disorder	Increase	ND	ND		T	Y	Linear, Focal	Fibrosis inflammatory cell infiltration	2	ND	Decompression surgery Glucocorticoid	Improved
2014	Zwicker et al.	Female	58	ND	Pain in the periscapular region	Pain in the periscapular region, Limb weakness, numbness and spasm	Normal	ND	ND		C T	Y	Linear	Inflammatory cell infiltration phlebitis	11.2	24	Decompression surgery Glucocorticoid Methotrexate	Improved
2016	Radotra et al.	Male	50	24w	Limb weakness	Limb weakness, Muscle atrophy, Urinary dysfunction	ND	ND	ND		L	Y	Focal	Fibrosis inflammatory cell infiltration	120–130	50	Decompression surgery Glucocorticoid	Improved
2016		Male	19	48w	Doraslgia	Doraslgia, limb weakness	ND	ND	ND		L	Y	Focal	Fibrosis inflammatory cell infiltration phlebitis	140–150	40	Decompression surgery Glucocorticoid	Improved
2016	Rui Gu et al.	Male	43	2w	Cervicalgia	Cervicalgia, limb weakness and loss of sensation, Urinary and fecal disorders	ND	ND	ND		C T	ND	ND	Fibrosis inflammatory cell infiltration	>50	>40	Decompression surgery Glucocorticoid	Improved
2016	Zhang Lu et al.	Male	55	24w	Limb numbness and weakness	Limb numbness and weakness, Urinary and fecal disorders	Normal	120	Increase	417.5	C T	Y	Linear	Fibrosis inflammatory cell infiltration	>10	>40	Decompression surgery Glucocorticoid Cyclophosphamide	Improved
2016	Ferreira et al.	Female	57	120w	Doraslgia	Doraslgia, limb numbness and weakness	Normal	ND	ND		T	Y	ND	Fibrosis inflammatory cell infiltration	ND	>50	Decompression surgery Glucocorticoid	Improved
2016	Yangue et al.	Male	18	ND	Paresthesia	Limb weakness, paresthesia, Balance disorder	ND	28	Increase	ND	B C	Y	Linear	Fibrosis inflammatory cell infiltration	ND	ND	Decompression surgery Glucocorticoid	Improved
2016	Mohanasundaram et al.	Female	34	ND	Limbs numbness and weakness, autonomic nerve dysfunction	Limbs numbness and weakness, Autonomic nerve dysfunction	Increase	0	Normal	ND	T	Y	Linear, Focal	Fibrosis inflammatory cell infiltration	ND	ND	Decompression surgery Glucocorticoid Rituximab	Improved Recurrence
2017	Williams et al.	Female	46	16w	Cervicalgia	Cervicalgia, limb weakness and loss of sensation	Normal	ND	ND		C T	Y	Linear	Fibrosis inflammatory cell infiltration phlebitis	10	ND	Glucocorticoid Azathioprine	Improved
2017	Qian Zhao	Female	49	4w	Doraslgia	Doraslgia	ND	ND	ND		T	Y	Linear, Focal	Fibrosis inflammatory cell infiltration	>100	ND	Decompression surgery	ND
2017	Rumalla et al.	Male	50	12w	Doraslgia	Doraslgia, dyspnea	Normal	ND	ND		T	Y	Focal	Fibrosis inflammatory cell infiltration	ND	ND	Decompression surgery Glucocorticoid	Improved
2017	Fernandez Codina et al.	Male	55	ND	Headaches	Headaches, decline in vision acuity, difficulty in swallowing, Scapular paralysis	Increase	43	Increase	ND	B C	Y	Linear	Fibrosis inflammatory cell infiltration	180	ND	Glucocorticoid Rituximab	Improved
2017	Maher et al.	Female	79	2w	Delirium, slurred speech, doraslgia.	Delirium, slurred speech, doraslgia.	Increase	Increase	Increase	ND	C T L	Y	Linear	Fibrosis inflammatory cell infiltration phlebitis	27	ND	Decompression surgery Glucocorticoid Rituximab	Improved Death
2018	Winkel et al.	Female	48	8w	Doraslgia	Doraslgia Intermittent claudication	Normal	ND	ND		L	Y	Focal	Fibrosis inflammatory cell infiltration	ND	27%	Decompression surgery Glucocorticoid	Improved
2018	Varrassi et al.	Male	62	ND	Headaches	Headaches, Decline in vision acuity, ptosis of the eyelids, Facial sensory disturbances, and limb weakness	Normal	Normal	Increase	162	C T L S	Y	Linear	Inflammatory cell infiltration	ND	ND	Glucocorticoid Rituximab	ND
2019	Sireesha et al.	Female	40	2w	ND	ND	Normal	ND	ND		C T	ND	ND	Fibrosis inflammatory cell infiltration phlebitis	ND	ND	Glucocorticoid	Improved
2019	Slade et al.	Male	50	20w	Doraslgia	Doraslgia, limb weakness and loss of sensation	Normal	ND	ND		C T	ND	ND	Fibrosis inflammatory cell infiltration	29	ND	Glucocorticoid Rituximab	Improved
2019	Levraut et al.	Male	55	ND	ND	ND	Normal	69	Increase	196	C T	Y	Linear nodular	Fibrosis inflammatory cell infiltration phlebitis	53	>40	Decompression surgery Glucocorticoid	Improved
2019	Merza et al.	Female	60	4w	Limbs weakness and numbness	Limbs weakness and numbness, Urinary and fecal disorders, cervicalgia	Increase	ND	ND		T	Y	Linear	Fibrosis inflammatory cell infiltration	ND	47	Decompression surgery Glucocorticoid	Improved
2019	Bridges et al.	Male	68	144w	Doraslgia	Doraslgia, limb numbness, Balance disorder	ND	ND	ND		T	Y	Focal	Fibrosis inflammatory cell infiltration	100	40	Decompression surgery Glucocorticoid	Improved
2020	Vakrakou et al.	Female	17	1w	Limbs numbness and paresthesia	Loss of sensation and limb weakness, Urinary and fecal disorders and cervicalgia	Increase	Normal	Normal	ND	C	Y	ND	Nd	ND	ND	Glucocorticoids Andazathioprine	Improved Recurrence
2020	Sbeih et al.	Male	24	192w	Doraslgia	Doraslgia, Limb numbness and weakness	Increase	ND	ND		C T	Y	Linear	Fibrosis inflammatory cell infiltration phlebitis	40	67	Decompression surgery Glucocorticoid	Improved
2020	Alrashdi et al.	Male	36	16w	Cervicalgia	Cervicalgia, limb weakness, Numbness in the shoulder	Increase	20	Increase	51	B C T	Y	Linear	Fibrosis inflammatory cell infiltration	ND	ND	Glucocorticoids Rituximab Methotrexate	Improved
2020	Zhang et al.	Male	39	2w	Doraslgia	Doraslgia, limb weakness, Urinary dysfunction	Normal	31	Increase	270	C T	Y	Linear	Fibrosis inflammatory cell infiltration	10	ND	Decompression surgery Glucocorticoid	Improved
2020	Hong-xing Li	Female	58	2w	Doraslgia	Doraslgia Urinary and fecal disorders, Sensory and motor disorders of the limbs	Increase	ND	ND		T	Y	Linear	Fibrosis inflammatory cell infiltration	>10	>40	Decompression surgery Glucocorticoid Cyclophosphamide	Improved Recurrence Death
2021	Sharma et al.	Male	27	144w	Vision decline	Decline in vision acuity, Limb numbness and weakness	Normal	ND	ND		B C	Y	Linear nodular	Fibrosis inflammatory cell infiltration	>10	60	Decompression surgery Glucocorticoid Azathioprine Rituximab	Improved
2021	Elmaci et al.	Female	37	ND	Cervicalgia and doraslgia, Hands weakness and numbness	Cervicalgia and doraslgia, numbness and weakness in the limbs	Normal	ND	ND		C T	Y	Linear	Fibrosis inflammatory cell infiltration	ND	ND	Decompression surgery	Improved Recurrence
2021	Sankowski et al.	Male	56	ND	Cervicalgia and gait disorder	Cervicalgia, cranial nerve palsy, Gait disorder	ND	ND	ND		C	Y	Linear	Inflammatory cell infiltration phlebitis	>10	40-50	Biopsy	ND
2021	Woo et al.	Male	62	4w	Headaches	Headaches, Cervicalgia, Limb weakness	Increase	40	Increase	180	B C	Y	Linear Focal	Inflammatory cell infiltration phlebitis	32	80	Decompression surgery Glucocorticoid Azathioprine.	Improved
2022	Karthigeyan et al.	Male	34	12w	Doraslgia	Doraslgia, limb weakness and Loss of sensation	Normal	ND	ND		T	Y	Focal	Fibrosis inflammatory cell infiltration	ND	ND	Decompression surgery Glucocorticoid Azathioprine	Improved
2022	Moskalik et al.	Female	34	288w	Headaches	headaches, tinnitus, limb weakness and loss of sensation, decline in vision acuity, dizziness, speech disorder	ND	ND	ND		B C T	Y	Linear	Fibrosis inflammatory cell infiltration	90	70	Decompression surgery Glucocorticoid	Improved Recurrence
2022	Kramer et al.	Female	35	24w	Cervicalgia Shoulder pain	Cervicalgia, Shoulder and arm pain	Normal	ND	ND		C T	Y	Focal	Fibrosis inflammatory cell infiltration	76	46	Decompression surgery Glucocorticoid	Improved
2022	Xia, C.; Li, P.	Female	61	48w	Limbs weakness and stiffness	Limbs weakness and stiffness Doraslgia, Defecation disorder	Increase	ND	ND		T	Y	Linear	Fibrosis inflammatory cell infiltration	ND	50	Decompression surgery	No improvement
2022	Yang et al.	Male	68	4w	Llimbs numbness and paresthesia, gait disorder	Limb numbness and weakness, Gait disorder, urinary dysfunction, Paraplegia	ND	ND	ND		T	ND	ND	Fibrosis inflammatory cell infiltration	ND	ND	Decompression surgery Glucocorticoid	Improved
2022		Male	39	2w	Doraslgia	Doraslgia, limb weakness, Gait disorder, urinary dysfunction	Normal	ND	ND		C T	Y	ND	Fibrosis inflammatory cell infiltration	>10	>40	Decompression surgery Glucocorticoid	Improved
2022		Male	43	2w	Cervicalgia	Cervicalgia, limb weakness, Urinary and fecal disorders	ND	ND	ND		C T	ND	ND	Fibrosis inflammatory cell infiltration	ND	>40	Decompression surgery Glucocorticoid	Improved
2023	Tanaviriyachai et al.	Female	49	1w	Doraslgia Progressive weakness	Doraslgia, limb weakness, urinary dysfunction, AbNormal sensation	Increase	ND	ND		T	Y	Linear	Fibrosis inflammatory cell infiltration	>10	>40	Decompression surgery Glucocorticoid Methotrexate	Improved
2023		Female	72	14w	Doraslgia	Doraslgia, Gait disorder	Increase	ND	ND		T	Y	Linear	Fibrosis inflammatory cell infiltration	15-45	>40	Decompression surgery Glucocorticoid	Improved
2023	Segers et al.	Female	55	12w	Doraslgia and gait ataxia	Gait ataxia, Doraslgia	Normal	ND	ND		T	Y	Linear, nodular	Fibrosis inflammatory cell infiltration	>50	90	Decompression surgery Glucocorticoid Rituximab	Improved
2023	Argenti et al.	Female	66	8w	Neck stifness Limbs dysesthesias and weakness	neck stifness, limbs dysesthesias and weakness	Increase	ND	ND		C T	Y	Linear, nodular	Inflammatory cell infiltration	ND	>40	Biopsy Glucocorticoid Rituximab	Improved
2024	Kageyama et al.	Female	69	16w	Doraslgia	Doraslgia	Increase	ND	ND		T	Y	Focal	Fibrosis	ND	ND	Decompression surgery Glucocorticoid Rituximab Methotrexate Mycophenolate mofetil	Improved
2024		Male	67	24w	Thigh pain	Leg pain	Normal	78	Increase	637	L	ND	ND	Fibrosis inflammatory cell infiltration phlebitis	ND	>40	Decompression surgery Glucocorticoid Azathioprine.	Improved
2024	Araújo et al.	Female	40	ND	Imbalance and shuffling gait	Gait disorder, Limb weakness, Difficulty in swallowing	ND	Normal	Normal		C T L	Y	nodular	Fibrosis inflammatory cell infiltration	ND	ND	Decompression surgery Glucocorticoid Cyclophosphamide Azathioprine Rituximab	Improved Death
2024	Chau et al.	Male	27	3w	Limb weakness and numbness	Limb weakness and numbness	Normal	ND	ND		C T	Y	Focal	Fibrosis inflammatory cell infiltration	20	90	Decompression surgery Glucocorticoid	Improved Recurrence
2024	Feldmann et al.	Female	65	16w	Pain in the left arm	The left arm is painful and numb, Gait disorder	Normal	125	Increase	3,260	C T	Y	Linear	Inflammatory cell infiltration	ND	>50	Decompression surgery Rituximab	Improved

Time, from onset to diagnosis; Y, yes; N, no; ND, not describe; B, brain; C, Cervical spine, T, Thoracic spine; L, Lumbar spine; S, Sacral spine.

## 3 Statistical analysis

The data were analyzed using SPSS 26.0 statistical software. The measurement data approximately conforming to a normal distribution and a skewed distribution were expressed as mean ± standard deviation (x ± s) and median (the first quartile, the third quartile) [M(Q1, Q3)], respectively. A *p*-value of < 0.05 was considered statistically significant.

## 4 Results

### 4.1 General information

Of the 55 patients, 29 were men and 26 were women, resulting in a male-to-female ratio of approximately 1.1:1. The mean age at onset was 48.29 years, ranging from 17 to 79 years. For 44 patients, the time from symptom onset to the diagnosis of IgG4-RSP upon admission was 7.93 (range 0.50–6.00) months.

### 4.2 Initial and principal symptoms

The initial symptoms of 53 patients were recorded, and the incidence rates, from highest to lowest, were as follows: pain (71.7%, 38/53), sensory abnormalities or weakness (32.1%, 17/53), gait disorders (7.5%, 4/53), and other symptoms such as difficulty swallowing and visual impairment. Among the patients whose initial symptom was pain, 47% (18/38) experienced dorsalgia, 21.1% (8/38) experienced cervicalgia, and the remaining patients experienced headache, limb pain, and scapular region pain, in that order. Among the 53 patients, the most common symptoms were sensory abnormalities or weakness (75.5%, 40/53) and pain (75.5%, 40/53). The other symptoms included urinary and fecal disorders (28.3%, 15/53), gait disorders, and cranial nerve involvement. Furthermore, 17% (9/53) of the patients experienced involvement of other organ systems.

### 4.3 Imaging manifestations

All 55 patients underwent at least one spinal magnetic resonance imaging (MRI) scan. The images revealed focal or diffuse thickening of the dura mater. A total of 49 patients were evaluated for enhanced MRI information, and of them, 98.0% (48/49) exhibited lesion enhancement on the enhanced MRI. Enhanced lesions were observed in 43 patients, including 29 cases of diffuse linear enhancement, 14 cases of focal or nodular enhancement, and 8 cases exhibiting both diffuse linear and focal or nodular enhancements. All spinal cord segments could be involved; the highest incidence was in the thoracic spine (*n* = 41, 74.5%), followed by the cervical spine (*n* = 31, 56.4%), lumbar spine (*n* = 10, 18.2%), and sacral spine (*n* = 2, 3.6%). Combined involvement of the cervical and thoracic spines was relatively common (*n* = 22, 40%). In addition, dura mater involvement in the brain was observed in 10 patients.

### 4.4 Laboratory tests

Among the 55 patients, serum IgG4 levels were measured in 39 patients, of whom 41.0% (16/39) had elevated serum IgG4 levels. Of the 17 patients who underwent routine cerebrospinal fluid examinations, cell count and protein levels in the cerebrospinal fluid increased in 70.6% (12/17) and 82.4% (14/17), respectively. The cerebrospinal fluid cell count ranged from 1 × 106 to 150 × 106, with a median of 31 × 106. The cerebrospinal fluid protein level ranged from 44 mg/dL to 865 mg/dL, with a median of 188 mg/dL.

### 4.5 Pathological examination

Of the 55 patients, 53 underwent pathological biopsy. The pathological changes were primarily characterized by lymphocyte and plasma cell infiltration. In addition, 86.8% (46/53) of the patients exhibited mat fibrosis, while 22.6% (12/53) exhibited obliterated phlebitis. Furthermore, 94.7% (36/38) had an IgG4-to-IgG cell ratio of more than 40% or more than 10 IgG4/high-power field (HPF) in pathological tissue.

### 4.6 Treatment and prognosis

A total of 98.2% (54/55) of the patients received at least one treatment protocol. Among them, 77.8% (42/54) underwent decompression surgery, 88.9% (48/54) received glucocorticoids, 25.9% (14/54) received traditional immunosuppressants (including azathioprine, methotrexate, and cyclophosphamide), and 22.2% (12/54) received rituximab. Furthermore, 40% (22/54) of the patients received a treatment regimen combining glucocorticoids with immunosuppressants and/or rituximab. Of the 55 patients, four were lost to follow-up and three died, two of whom died from secondary infections following glucocorticoid and immunosuppressant treatment. Six cases had symptom recurrence, and 98.0% (50/51) of the patients experienced varying degrees of symptom relief.

## 5 Discussion

IgG4-related disease (IgG4-RD) is an inflammatory and fibrotic disorder that affects multiple organs. It is mainly characterized by elevated serum IgG4 levels and the enlargement of affected organs and tissues. The most frequently affected glands include the pancreas, submandibular gland, and lacrimal gland. Involvement of the nervous system is rare (Wallace et al., 2019). In the nervous system, the dura mater and pituitary gland are the most frequently affected sites. Previous studies have statistically shown that the incidence of IgG4-related pachymeningitis is 1.9% (Wallace et al., 2015), while the incidence of hypophysitis is between 1.7% and 2.3% (Zhang et al., 2017; Lin et al., 2015). However, IgG4-RSP is primarily reported as individual cases. This disease primarily affects middle-aged individuals, with a male-to-female ratio of approximately 1.1:1. The clinical manifestations are complex and diverse, posing significant challenges to diagnosis. This study summarizes the clinical and pathological characteristics of patients with IgG4-RSP to enhance neurologists' awareness of this disease and enable early diagnosis and treatment.

The clinical manifestations of IgG4-RSP are non-specific and associated with the affected spinal cord regions. They mainly manifest as radicular pain and symptoms of spinal cord compression. Among the 53 cases described in this article, 38 patients reported pain as their initial symptom. Of those patients, 47% experienced dorsalgia and approximately 21% experienced cervicalgia. The most prevalent symptoms were weakness and/or sensory abnormalities in the trunk or limbs (75.5%), pain in the trunk or limbs (75.5%), and urination and bowel disorders (28.3%).

The symptoms of IgG4-RSP are attributed to compression of the spinal nerve roots and spinal cord by thickened and hyperplastic dura mater. Spinal cord imaging examinations are essential for diagnosis. Magnetic resonance imaging (MRI) of the spinal cord is the preferred diagnostic method for IgG4-RSP because it can accurately display the lesion's location, range, and degree of spinal cord compression, as well as its progression during follow-up. The condition is characterized by uneven thickening of the affected dura mater, which appears as a low signal on T1WI images and a low or isointense signal on T2WI images. Cross-sectional images show that the thickened dura mater compresses the adjacent spinal cord, causing it to become thinner. An enhanced scan reveals dura mater enhancement (Alsulaiman, 2020). In the patients with IgG4-RSP summarized in this article, spinal cord involvement most commonly occurred in the thoracic spine, followed by the cervical spine. This finding is consistent with previous studies (Yang et al., 2022). If a patient exhibits symptoms of spinal cord compression and MRI of the spinal cord shows the aforementioned manifestations, the possibility of IgG4-RSP should be considered. Additional examinations should be performed to aid in the diagnosis.

There are no separate diagnostic criteria for IgG4-RSP. Diagnosis primarily depends on the diagnosis of IgG4-related disease and involvement of the spinal dura mater. The Comprehensive Diagnostic Criteria for IgG4-Related Diseases (2011) (Umehara et al., 2012), established in Japan in 2011, were the earliest comprehensive diagnostic guidelines for IgG4-RD. They encompass three aspects: clinical manifestations, serum IgG4 levels, and pathological features. The criteria were updated and discussed in 2020 (Umehara et al., 2021). Simple lymph node enlargement does not meet the requirements for clinical and imaging features. Typical tissue fibrosis, especially mat fibrosis, and obliterative phlebitis have been added to the pathological diagnostic features. Serum IgG4 levels are elevated in up to 90% of patients with IgG4-RD, but this estimate varies substantially depending on the type of patients included in studies (Katz and Stone, 2022). A 2016 Japanese survey revealed that 84.5% of patients with type 1 autoimmune pancreatitis (AIP) had high serum IgG4 levels (Masamune et al., 2020). Alessia Buglioni's research demonstrated that serum IgG4 levels were increased in 81% of patients with IgG4-related kidney disease (IgG4-RKD) (Buglioni et al., 2024). Xia et al. (2023) recruited a cohort of 40 patients diagnosed with IgG4-related sialadenitis (IgG4-RS), all exhibited elevated serum IgG4 levels. While elevated serum IgG4 levels are important for diagnosing IgG4-RD, some patients with IgG4-RSP do not exhibit elevated levels. The case results summarized in this article showed that approximately two-thirds of the patients had normal serum IgG4 levels, which is consistent with previous findings (Lu et al., 2016).

Therefore, elevated serum IgG4 levels are not specific to the diagnosis of IgG4-RD. However, clinical studies have shown (Wallace et al., 2015; Lin et al., 2015) that serum IgG4 levels can reflect disease activity to some extent and are positively correlated with the number of affected organs and the degree of organ fibrosis. In 2019, the American College of Rheumatology (ACR) and the European League Against Rheumatism (EULAR) jointly introduced the first international classification standard for IgG4-RD (Wallace et al., 2020), emphasizing typical clinical manifestations of affected organs and changes observed in laboratory and imaging studies. This standard assigns weighted scores to each assessment indicator. This addresses the issue of diagnosing IgG4-RD when serum IgG4 levels are normal and pathological results are lacking. It is also more applicable to patients with IgG4-RD involving multiple organs. A diagnosis of IgG4-RSP still depends on pathological alterations. The characteristic histological features of IgG4-RD are as follows: (1) massive lymphoplasmacytic infiltration, (2) mat fibrosis, and (3) obliterative phlebitis (Deshpande et al., 2012). For immunoglobulin G4-related hypertrophic pachymeningitis (IgG4-RHP), immunohistochemical staining requires more than 10 IgG4-positive plasma cells per high-power field (HPF), and the IgG4-positive/IgG-positive plasma cell ratio must be greater than 40% (Saitakis and Chwalisz, 2021). The pathological diagnosis of IgG4-RSP mainly refers to that of IgG4-RHP.

IgG4-RD is an immune-mediated, chronic, inflammatory disease. Currently, drug treatment is based on glucocorticoids, supplemented by immunosuppressants and even biological agents (Perugino and Stone, 2020). When IgG4-RSP causes progressive neurological symptoms due to spinal cord compression, surgical decompression is effective. Patients with IgG4-RD respond well to glucocorticoids (Seegobin et al., 2021), but there are no clear standards for the dosage, tapering schedule, or duration of maintenance treatment. Clinical experience is the main guide for treatment. While nearly all patients with IgG4-RD respond to glucocorticoids, approximately 40% do not achieve complete remission and relapse within 1 year (Yunyun et al., 2017). When used in combination with glucocorticoids, immunosuppressants such as methotrexate, cyclophosphamide, azathioprine, tacrolimus, and mycophenolate mofetil can improve outcomes, reduce recurrence, accelerate the taper process, and reduce glucocorticoid side effects (de Pretis et al., 2017; Della-Torre et al., 2015; Luo et al., 2020; Buechter et al., 2014). If the aforementioned treatments are ineffective, biological agents can be considered. Rituximab (RTX) is the first and most widely used biological agent for treating IgG4-RD. It can significantly reduce serum IgG4 levels (Khosroshahi et al., 2010). RTX has a significant therapeutic effect on IgG4-RD remission (Carruthers et al., 2015), and regular use can reduce IgG4-RD recurrence (Campochiaro et al., 2019). Of the 55 patients discussed in this article, 48 received glucocorticoids, 14 received immunosuppressants, and 12 received rituximab. While the majority of the patients showed short-term improvement, no long-term improvement was observed, and some experienced recurrence during follow-up.

### 5.1 Limitations

As the dataset was based on case reports and small-scale studies, there may be inconsistencies in the descriptions of clinical symptoms, laboratory and imaging findings, and treatment plans.

## 6 Conclusion

IgG4-related spinal pachymeningitis (IgG4-RSP) is relatively uncommon in clinical settings. Its clinical symptoms and imaging results are not specific and depend on the affected area. Symptoms include local pain and spinal cord compression, which can result in progressive neurological impairment and paralysis. In patients with normal serum IgG4 levels and no other systemic involvement, the possibility of IgG4-RSP should be considered, and a dural biopsy should be performed promptly. Once the diagnosis is confirmed, early treatment is necessary; the prognosis for most treated patients is relatively good. The gold standard for diagnosing the disease is a spinal cord biopsy, but obtaining the sample is difficult and highly invasive. Some scholars have proposed using the quantitative concentration of IgG4 and the IgG4 index in cerebrospinal fluid as an alternative to biopsy. However, there are only a few case reports on IgG4 concentrations in cerebrospinal fluid, so its clinical application is limited. This may serve as a potential direction for future studies.

## Data Availability

Publicly available datasets were analyzed in this study. This data can be found here: no.
